# Supplementary material for: COZOID: contact zone identifier for visual analysis of protein-protein interactions
Source: BMC Bioinformatics. 2018 Apr 6;19:125. doi: 10.1186/s12859-018-2113-6 (PMC5889581; doi:10.1186/s12859-018-2113-6)
Supplement: Supplementary file 2 — Software build. Executable binary file of the software tool. (ZIP 118,784 kb) [file 12859_2018_2113_MOESM2_ESM.zip › cozoid/java/docs/beansbinding-1.2.1-doc.zip!/org/jdesktop/el/package-tree.html]

org.jdesktop.el Class Hierarchy (Beans Binding)


---


|  |  |  |
| --- | --- | --- |
| **Overview** \| **Package** \| Class \| Use \| **Tree** \| **Deprecated** \| **Index** \| **Help** | |  |
| **PREV**   **NEXT** | **FRAMES**    **NO FRAMES**     **All Classes** |  |


---


## Hierarchy For Package org.jdesktop.el

**Package Hierarchies:**: All Packages

---

## Class Hierarchy

- java.lang.Object
  - org.jdesktop.el.**BeanELResolver.BeanProperties**- org.jdesktop.el.**BeanELResolver.BeanProperty**- org.jdesktop.el.**ELContext**- org.jdesktop.el.**ELResolver**
          - org.jdesktop.el.**ArrayELResolver**- org.jdesktop.el.**BeanELResolver**- org.jdesktop.el.**CompositeELResolver**- org.jdesktop.el.**ListELResolver**- org.jdesktop.el.**MapELResolver**- org.jdesktop.el.**ResourceBundleELResolver**
          - java.util.EventObject (implements java.io.Serializable)
            - org.jdesktop.el.**ELContextEvent**
            - org.jdesktop.el.**Expression** (implements java.io.Serializable)
              - org.jdesktop.el.**MethodExpression**- org.jdesktop.el.**ValueExpression**
              - org.jdesktop.el.**Expression.ResolvedProperty**- org.jdesktop.el.**Expression.Result**- org.jdesktop.el.**ExpressionFactory**- org.jdesktop.el.**FunctionMapper**- org.jdesktop.el.**MethodInfo**- java.lang.Throwable (implements java.io.Serializable)
                          - java.lang.Exception
                            - java.lang.RuntimeException
                              - org.jdesktop.el.**ELException**
                                - org.jdesktop.el.**MethodNotFoundException**- org.jdesktop.el.**PropertyNotFoundException**- org.jdesktop.el.**PropertyNotWritableException**
                          - org.jdesktop.el.**VariableMapper**

## Interface Hierarchy

- java.util.EventListener
  - org.jdesktop.el.**ELContextListener**

## Enum Hierarchy

- java.lang.Object
  - java.lang.Enum<E> (implements java.lang.Comparable<T>, java.io.Serializable)
    - org.jdesktop.el.**Expression.Result.Type**

---


|  |  |  |
| --- | --- | --- |
| **Overview** \| **Package** \| Class \| Use \| **Tree** \| **Deprecated** \| **Index** \| **Help** | |  |
| **PREV**   **NEXT** | **FRAMES**    **NO FRAMES**     **All Classes** |  |


---
